# Energy-harvesting materials based on the anomalous Nernst effect

**DOI:** 10.1080/14686996.2019.1585143

**Published:** 2019-03-26

**Authors:** Masaki Mizuguchi, Satoru Nakatsuji

**Affiliations:** a Institute for Materials Research, Tohoku University, Sendai, Japan; b CREST, Japan Science and Technology Agency, Kawaguchi, Japan; c Center for Spintronics Research Network, Tohoku University, Sendai, Japan; d The Institute for Solid State Physics, The University of Tokyo, Kashiwa, Japan; e Institute for Quantum Matter and Department of Physics and Astronomy, Johns Hopkins University, Baltimore, MD, USA

**Keywords:** Energy harvesting, thermoelectric conversion, Nernst effect, anomalous Nernst effect, Seebeck effect, spin Seebeck effect, magnetic anisotropy, spin-orbit interaction, Weyl magnet, 50 Energy Materials, 106 Metallic materials, 203 Magnetics / Spintronics / Superconductors

## Abstract

The anomalous Nernst effect (ANE), one of the thermomagnetic effects studied for a long time, has recently attracted renewed attention. The ANE, which originates from fictitious fields in momentum space, is essential for clarifying the interplay among heat, spin, and charge in magnets. Moreover, compared to the Seebeck effect, it has various benefits for application to high-efficiency energy-harvesting devices as it may provide much more simple lateral structure, higher flexibility, and much lower production cost. In this review, we discuss various topics related to the methods to modulate the ANE for its thermoelectric applications. In addition, we review strategies to design materials to obtain large ANE including Weyl magnets and thermoelectric devices for effectively utilizing the ANE.

## Graphical Abstract

**Figure:**
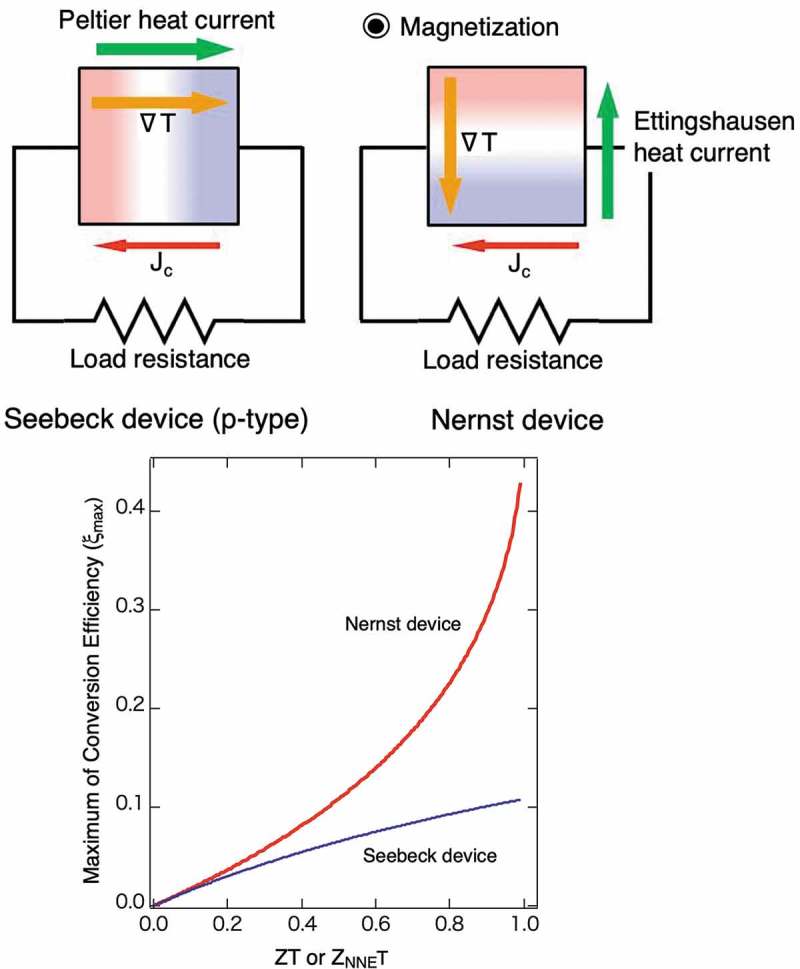

## 1. Introduction

In the advanced Internet of Things (IoT) society in the near future, the energy harvesting is a key technology to control energy, through accumulation, storage, and use of power. Several small-scale ambient energy sources such as heat, light, electromagnetic waves, and mechanical vibration are proposed to be useful for energy-harvesting technology. Thermoelectric generation is a popular method to generate electricity because it is a clean conversion using not only waste heat from such as households, motor vehicles, and factories but also heat from naturally abundant, environmental heat sources. Especially, as we discuss below, it is a promising and challenging strategy to use magnetism in thermoelectric conversion.

**Figure 1.**
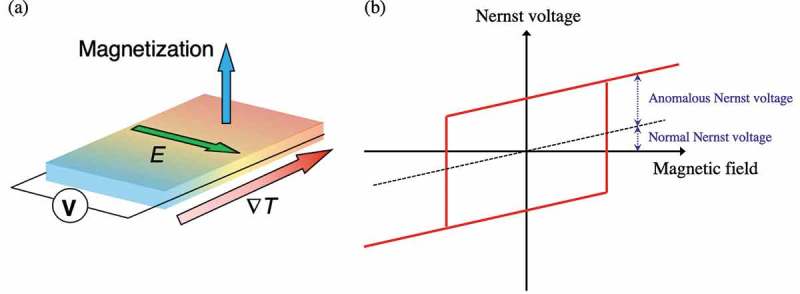
Schematic images of (a) the anomalous Nernst effect and (b) hysteresis of the Nernst voltage as a function of magnetic field.

Recently, the research field covering spintronics and thermoelectrics, that is, ‘spin caloritronics’, has attracted significant attention [1–7]. The thermoelectric conversion from heat to electric energy via spin has proved promising for further developing the energy-harvesting technology because spin can be controlled by quite a small energy in nanostructures. Traditionally, the Seebeck effect is known as a representative thermoelectric effect and widely used in numerous nonmagnetic thermoelectric devices. This effect directly converts heat into electricity; electric power can be created along the direction of temperature gradient. On the other hand, the Nernst effect is another well-known thermoelectric effect [8]. When the temperature gradient (∇*T*) and the magnetic field (*H*), which are normal to each other, are applied to a conductor, an electromotive force is induced normal to both of ∇*T* and *H,* and a Nernst voltage can be observed. In addition, if the material has a spontaneous magnetization, spontaneous term of the Nernst effect becomes superimposed on the normal Nernst term as shown in Figure 1(a). This spontaneous term is called the anomalous Nernst effect (ANE) and frequently observed in ferromagnetic materials as shown in Figure 1(b) [6,7,9–14]. An observable electric field (*E*) is described as

(1) $$E=E_{NNE}+E_{ANE}=Q_{0}(H\times \nabla T)+Q_{s}(\mu_{0}M_{s}\times \nabla T)$$

where **E**_NNE_, **E**_ANE_, *Q*_0_, *Q*_s_, *μ*_0_, and **M** *_s_* refer to the normal Nernst electric field vector, the anomalous Nernst electric field vector, the Nernst coefficient, the anomalous Nernst coefficient, vacuum permeability, and magnetization vector, respectively. The normal Nernst effect is proportional to **H**, and hence enough magnetic field is always necessary for the thermoelectric conversion. In contrast, the ANE is spontaneous at zero field and proportional to saturation magnetization in principle. Recent experimental and theoretical studies have revealed that it originates from the fictitious field (Berry curvature) in the momentum space in magnets and can be particularly enhanced when the Weyl points are tuned to be close to the Fermi energy as we will discuss in the section for the Weyl magnets [7]. If magnetic materials with large *Q*_s_ and/or ***M*** *_s_* are developed and the remanence state is used, ANE is expected without applying a magnetic field. This distinctive feature of the ANE appears suitable for the thermoelectric conversion process. However, there have been no reports on the development of actual devices based on the Nernst effect, nor the ANE.

In this article, we review the recently developed energy-harvesting materials based on the ANE and the demonstration of the proof-of-concept for the application of the ANE to thermoelectric conversion devices. First, benefits for using the ANE-based thermoelectric conversion compared with the Seebeck-based counterpart are discussed in Section 2. The advantage in generating efficiency of the ANE-based thermoelectric conversion is numerically examined. Next, characteristics of the ANE in several ordered-alloys thin films are systematically investigated to establish the guideline for an energy-harvesting application [15] in Section 3. The relationship between magnetic anisotropy and the ANE is discussed for ordered alloys. As one of the practical application methods using the ANE, an ANE-based thermopile system is proposed and experimentally demonstrated, revealing high degrees of freedom in designing thermopile systems [16,17] in Section 4. New trends in the development of prominent materials for the ANE and energy-harvesting thermoelectric applications are also presented in Section 5 [18–22]. Novel strategies to effectively enhance and control the ANE are reviewed. In particular, we highlight the large ANE observed in an antiferromagnetic bulk with a very small magnetization in Section 6. This interesting phenomenon arises from an enhanced Berry curvature coming from Weyl points near the Fermi energy. Finally, we discuss the new strategy to develop the Weyl magnets as a new class of thermoelectric materials [7,23].

## 2. Advantages of ANE-based energy-harvesting thermoelectric conversion system

Before reviewing several experiments, a comparison between the performance of the Nernst effect and the Seebeck effect regarding the thermoelectric conversion is discussed in this section, and an advantage of the ANE-based energy-harvesting thermoelectric conversion system is shown. The Seebeck effect needs only temperature difference; it requires no mechanical motion, and thus is suitable for reliable and soundless conversion devices. The dimensionless figure of merit, *ZT*, is widely used to evaluate the performance of thermoelectric devices; it is defined as:

(2) $$ZT=\frac{\sigma \cdot S^{2}}{\kappa }T$$

where *T, σ*, and *κ* are temperature, the electrical conductivity, and the thermal conductivity, respectively. In general, the condition *ZT* > 1 is required for an effective application of the Seebeck effect, and this condition is somehow difficult to satisfy from a perspective of material design. The Nernst effect is also expected to be applied for the thermoelectric conversion. A decisive difference between the Nernst effect and the Seebeck effect is the output direction against the temperature gradient, that is, the Nernst effect is a transverse effect, whereas the Seebeck effect is a longitudinal one. This difference determines an inevitable performance of each effect.

**Figure 2.**
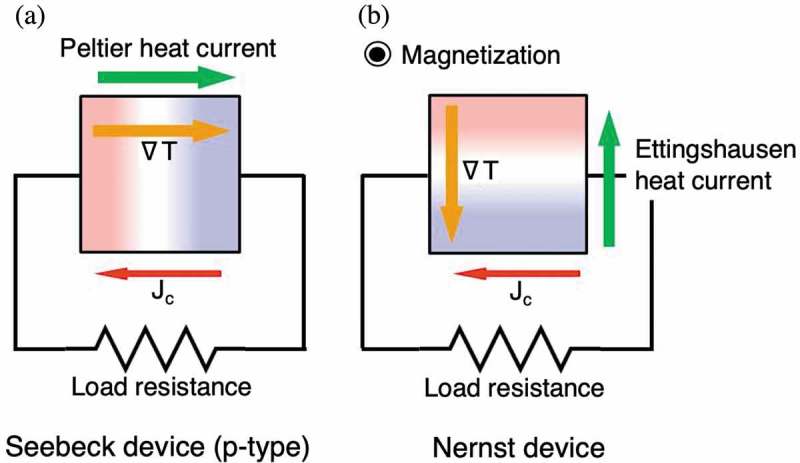
Schematic images of flows of energy in (a) the Seebeck device (p-type) and (b) the Nernst device during operation.

**Figure 3.**
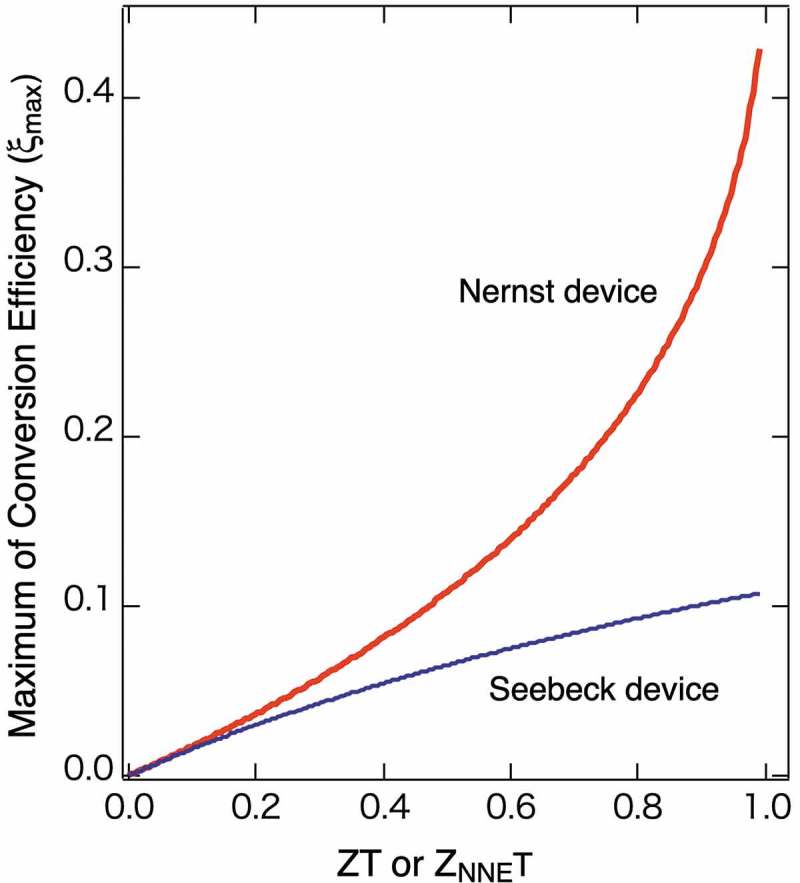
Calculated maximum of the conversion efficiency (*ξ*_max_) as a function of the adiabatic figure of merit (*ZT* or *Z*_NNE_ *T*), assuming that temperatures at the hot and cold side are 600 and 300 K, respectively.

Figure 2 depicts the schematic images of flows of energy in the Seebeck device and the Nernst device during operation. The temperature difference in both devices yields an electromotive force and electrical current (*J*_c_) flow. For the Seebeck device (p-type), Peltier heat current caused by *J*_c_ flows from the hot side to the cold side in the device, along the same direction as the temperature gradient. As a result, this Peltier heat current also conveys heat, and thus degrades the conversion efficiency. For the Nernst device, *J*_c_ brings the Ettingshausen heat current from the cold side to the hot side. This heat current helps to reinforce the generating efficiency in the Nernst device. Eventually, maximum of the conversion efficiency (*ξ*_max_) which ignores the Joule heating by the electrical current behaves differently with *J*_c_ for the two devices. Calculated *ξ*_max_ for both devices as a function of the figure of merit is shown in Figure 3, assuming that temperatures at the hot and cold side are 600 and 300 K, respectively [24]. Here, the figure of merit for the Nernst effect is defined as:

(3) $$Z_{NNE}T=\frac{\sigma_{yy}\cdot {Q_{0}}^{2}\cdot {H_{z}}^{2}}{\kappa_{xx}}T$$

Both *ξ*_max_ increase with *ZT* and *Z*_NNE_ *T* increase. Interestingly, *ξ*_max_ for the Nernst device apparently increases more rapidly than that of the Seebeck device. This calculation proves that in case having the same value for both *ZT* and *Z*_NNE_ *T, ξ*_max_ of the Nernst device becomes always larger than that of the Seebeck effect. This is one of the advantages of the Nernst effect, and the larger *Z*_NNE_ *T* becomes, this merit becomes more prominent. It should be noted that the argued *Z*_NNE_ *T* value here is an adiabatic *Z*_NNE_ *T*. This should be distinguished from an isothermal *Z*’_NNE_ *T* which has a maximum limit of *Z*’_NNE_ *T* = 1 [25].

Another notable advantage of the Nernst effect is that the observed Nernst voltage is governed by not the temperature difference but the temperature gradient. It is true that for the Seebeck effect as well, the voltage increases proportionally with the temperature gradient. However, a sufficient length of the material along the temperature gradient is indispensable to secure a large temperature difference, which decides the Seebeck voltage. As for the Nernst effect, if the temperature gradient is provided, no long length of the material along the temperature gradient is necessary because the Nernst voltage increases with the transverse length normal to both the temperature gradient and the magnetic field (the magnetization in the case of ANE). Thus, the Nernst effect is suitable for the thermoelectric conversion system composed of thin materials and therefore useful to design a flexible device to fit any type of curved surface of a heat source. This crucial advantage of the Nernst effect is also discussed in the section of ANE-based thermopile systems.

Moreover, a thermoelectric conversion module based on the Nernst effect has high degrees of freedom for designing because the Nernst voltage does not decrease due to the Ettingshausen heat current, whereas the Peltier heat current may affect the Seebeck voltage. This enables the multi-dimensional design of the Nernst-based module, and as a result, a larger scale integration of the module is much more plausible compared with the Seebeck-based one. In fact, as we discussed above, the ANE allows us to design a laterally connected structure, suitable to cover a large area of a heat source with much smaller numbers of fabrication processes than the commonly used 3D pillar structure of the Seebeck modules, thus reducing the production cost significantly. The (anomalous) Nernst-based module is compatible with the nanoscale constitutions fabricated by any microfabrication to the large-area, flexible elements such as thermoelectric sheets. As a reference, *ZT* for the anomalous Nernst effect is defined as:

(4) $$Z_{ANE}T=\frac{\sigma_{yy}\cdot {Q_{s}}^{2}\cdot {\left(\mu_{0}M_{z}\right)}^{2}}{\kappa_{xx}}T$$

Recently, the spin Seebeck effect has been found and stimulated spin caloritronic researches [3]. The effect is indeed innovating and interesting from a perspective not only of basic science but also of thermoelectric applications, such as thermoelectric sheets discussed above. However, a spin detection material with a strong spin-orbit interaction (SOI) such as Pt is necessary for the spin Seebeck effect because the thermally generated spin current is converted to voltage in the material. In addition, as this is an interface effect, the high interface resistance to create the large voltage tends to suppress the efficiency and thus requires a complicated, multi-layered structure to enhance the size of the effect. While *ZT* can be also defined for the spin Seebeck effect (*Z*_SEE_ *T* = 0.0004) [25], it is far smaller than those of the widely used Seebeck devices so far, which is the same situation of *ZT* for the Nernst effect (*Z*_ANE_ *T* is 0.000004 for Mn_3_Sn and 0.0008 for Co_2_MnGa) [26]. Hence, it is strongly desired that a revolutionary new material with a large Nernst effect is discovered, which will ultimately lead to the large enhancement in *ZT* for the Nernst effect.

## 3. Characteristics of ANE in ordered alloy thin films

In this section, characteristics of several ordered alloys thin films are systematically investigated to establish the guideline for an energy-harvesting application. Chemically ordered binary alloys composed of a 3*d* transition element and a noble metal, such as FePt, FePd, CoPt, and CoPd, show extremely large uniaxial magnetic anisotropy and coercivity [27–30]. In particular, an *L*1_0_-ordered (CuAu-type) FePt is known to have an extremely large magnetic anisotropy; the uniaxial magnetic anisotropy energy (*K*_u_) of this material has been reported to be 7.0 × 10^7 ^erg/cm^3^ [28]. The large magnetic anisotropy is attributed to the strong SOI in the Pt 5*d* orbitals. It is considered that the Fe atom induces a spin magnetic moment in the Pt atom through the hybridization between the Fe 3*d* and Pt 5*d* states, and the electronic structure of an *L*1_0_-ordered FePt film has been studied by hard X-ray photoelectron spectroscopy (HAXPES) and first-principles calculations [31]. An origin of the ANE is also attributed to the SOI; hence, it is essential to study the relationship between the SOI and ANE from both perspectives of a scientific principle and an energy-harvesting application. In this review, for the purpose of clarifying the relationship between *K*_u_ and the ANE, we discuss the ANE of epitaxially fabricated thin films of the ordered alloys with different *K*_u_, including *L*1_0_-ordered FePt, *L*1_0_-ordered FePd, *L*1_0_-ordered MnGa, and *D*0_22_-ordered (Al_3_Ti-type) Mn_2_Ga [15].

*L*1_0_-ordered FePt, *L*1_0_-ordered FePd, *L*1_0_-ordered MnGa, and *D*0_22_-ordered Mn_2_Ga thin films with a thickness of 30 nm are deposited on MgO(001) single crystal substrates using a sputtering method. The four samples are fabricated by employing conditions optimized to maximize *K*_u_. Measurements of the ANE are conducted in a physical properties measurement system with a superconducting magnet. ∇*T* was produced along the in-plane direction of the films by applying the heat at one side of each sample edge. The electric field across the temperature gradient (*E*_xy_) was measured under a magnetic field perpendicular to the film. In this review, the transverse Seebeck coefficient (*S*_xy_) is used as a parameter indicating the magnitude of the Nernst effect by defining as follows:

(5) $$S_{xy}\;=\;E_{xy\;}/\nabla T$$

**Figure 4.**
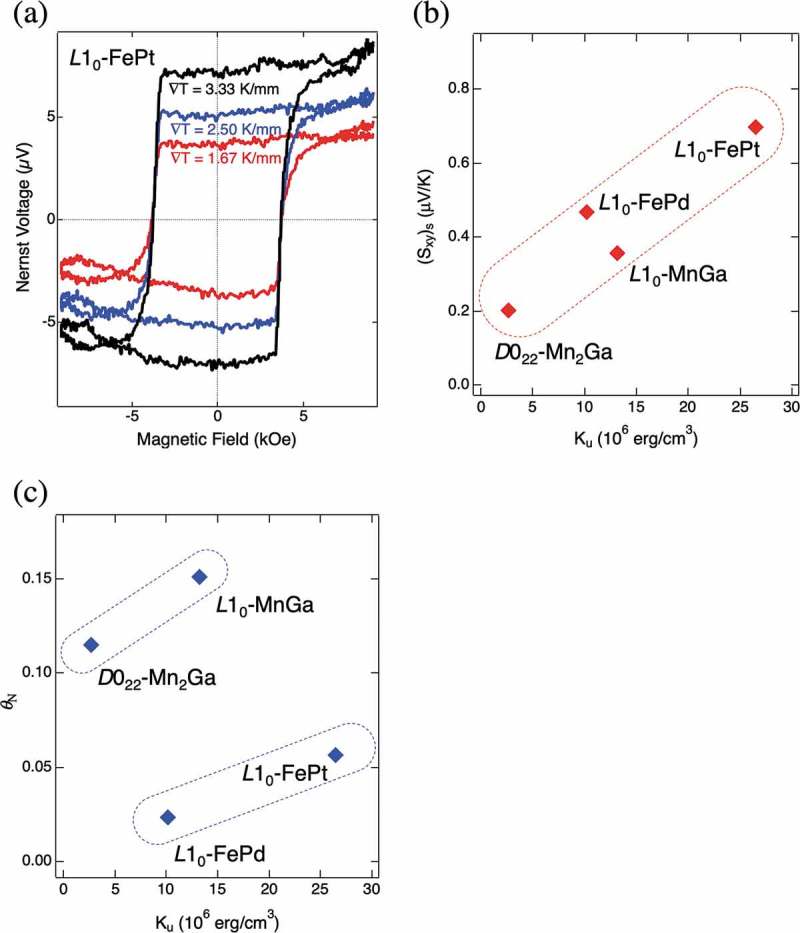
(a) The Nernst voltages for an *L*1_0_-ordered FePt thin film with the uniaxial magnetic anisotropy energy (*K*_u_) of 3.0 × 10^7^ erg/cm^3^ measured as a function of magnetic field with different temperature gradients (∇*T*) [6]. (b) Material dependence of the anomalous term of the transverse Seebeck coefficient ((*S*_xy_)_s_) at 300 K as a function of *K*_u_. (c) Material dependence of the Nernst angle (*θ*_N_) at 300 K as a function of *K*_u_ [15].

Figure 4(a) presents the Nernst voltages for an *L*1_0_-ordered FePt thin film with *K*_u_ of 3.0 × 10^7 ^erg/cm^3^ measured as a function of magnetic field. Clear indication of electromotive force is observed with a hysteresis. The shape of the hysteresis loop coincides with that of magnetization. ∇*T* is also changed for this measurement as shown in the figure, and it is confirmed that anomalous Nernst voltage increases proportionally with ∇*T*. These results demonstrate that the measured voltage is certainly caused by the anomalous Nernst effect in the FePt layer.

The ANE of other ordered alloy thin films are also measured and the anomalous term of *S*_xy_, that is, (*S*_xy_)_s_ measured at 300 K is plotted as a function of *K*_u_ in Figure 4(b). Overall, (*S*_xy_)_s_ increases monotonically with *K*_u_ regardless of the material. This implies that the ANE increases roughly with the strength of the SOI in the ordered alloys measured in this study. These results indicate the ordered materials with large magnetic anisotropy are suitable to obtain the large ANE.

Figure 4(c) shows the *K*_u_ dependence of the Nernst angle, the ratio between the Nernst and Seebeck effects, *θ*_N_ = *S*_xy_/*S*_xx_ for several ordered material thin films. It is clarified that *θ*_N_ of Mn-based alloys are comparatively larger than those of Fe-based ones. One of the reasons for the large *θ*_N_ of Mn-based ordered alloys is that their *S*_xx_ are relatively lower compared with Fe-based ordered alloys. This insists that *θ*_N_ is not directly related with *K*_u_, and thus it is important to choose adequate materials and parameters suitable for each ANE-based energy-harvesting device.

## 4. Demonstration of ANE-based thermopile systems

**Figure 5.**
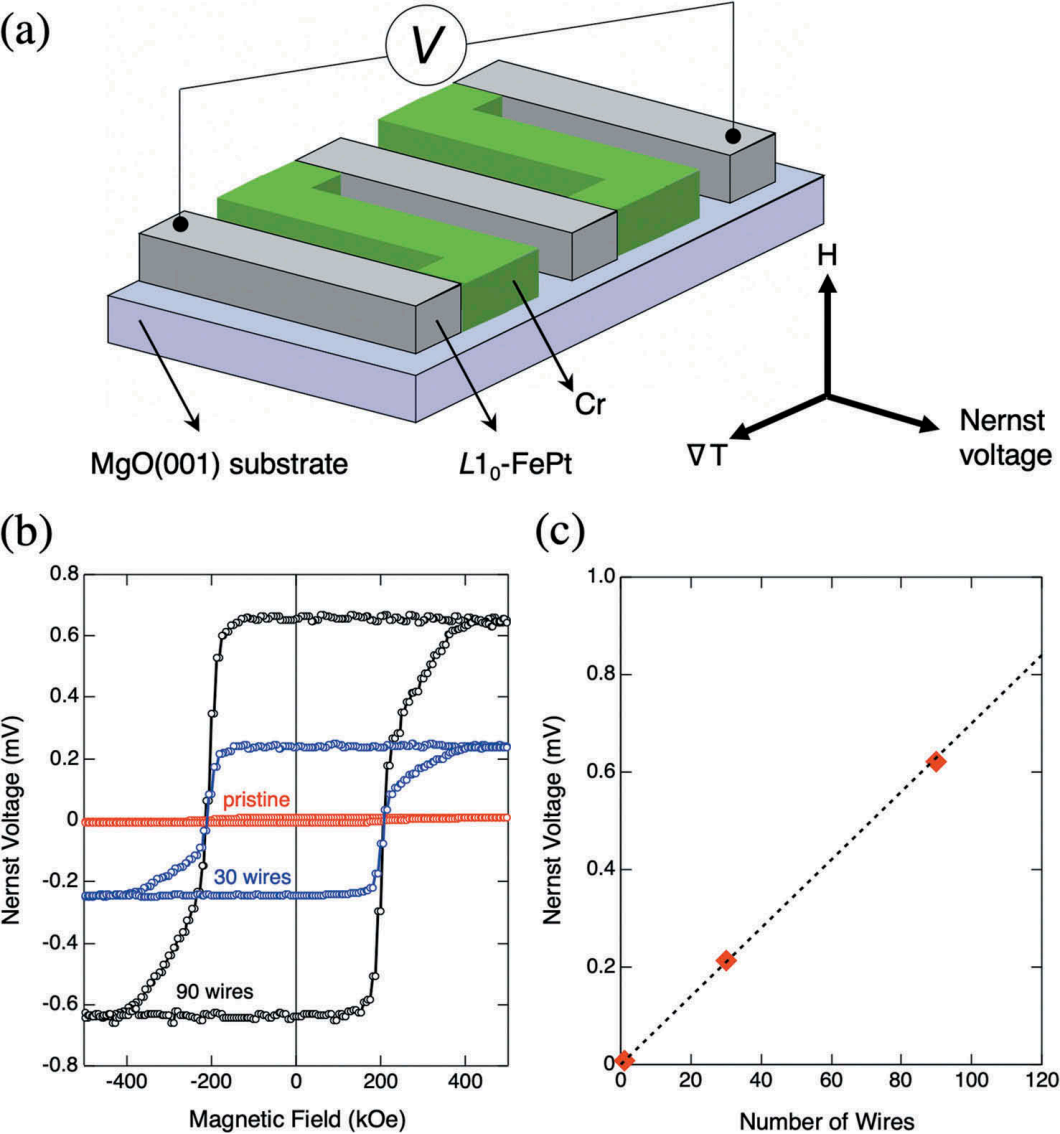
(a) The thermopile where ferromagnetic *L*1_0_-ordered FePt wires and nonmagnetic Cr wires are connected alternatively in series. Each wire has a size of 6 mm length, 5 μm width, and an interval of adjacent wires of 5 μm. An electromotive force between both edges of thermopile is measured as the Nernst voltage at room temperature with in-plane ∇*T* and out-of-plane *H*. (b) The Nernst voltage as a function of magnetic field with different numbers of FePt wires. (c) The ANE voltage as a function of the number of FePt wires [16].

To apply the ANE to an actual thermoelectric conversion device, the efficient and relevant designs of ANE-based systems are founded on the device-architecture. Here, an ANE-based thermopile system consisting of ferromagnetic wires is proposed and its proof of concept is demonstrated [16,17]. The thermopile is similar to the spin-Hall thermopile using the spin-Seebeck effect and is composed of the connected metallic wires in series [32]. Particularly in this review, we introduce the thermopile where ferromagnetic *L*1_0_-ordered FePt wires and nonmagnetic Cr wires are connected alternatively in series as shown in Figure 5(a). Each wire has a size of 6 mm length, 5 μm width, and an interval of adjacent wires of 5 μm. The number of connected FePt wires is varied, and an electromotive force between both edges of thermopile is measured as the Nernst voltage at room temperature. The Nernst voltages of a thermopile with 30 wires, that with 90 wires, and a pristine FePt film are measured as shown in Figure 5(b). A thickness of a pristine FePt film is 20 nm, and it is microfabricated into FePt wires. ∇*T* of 3.3 K/mm is applied along the in-plane direction, and magnetic field is applied perpendicular to the film. The ANE of both thermopiles is clearly larger, compared with that of the pristine FePt film. This demonstrates that the thermopile structure is substantially effective to enhance the ANE. In addition, the Nernst voltage of a thermopile with 90 wires is larger than that of 30 wires. Figure 5(c) is a plot of the Nernst voltage as a function of the number of wires. It is found that the Nernst voltage is proportional to the number of wires, and one FePt wire produces the voltage of 6.6 μV. This suggests that the ANE can be controlled by the proper design of a thermopile structure.

**Figure 6.**
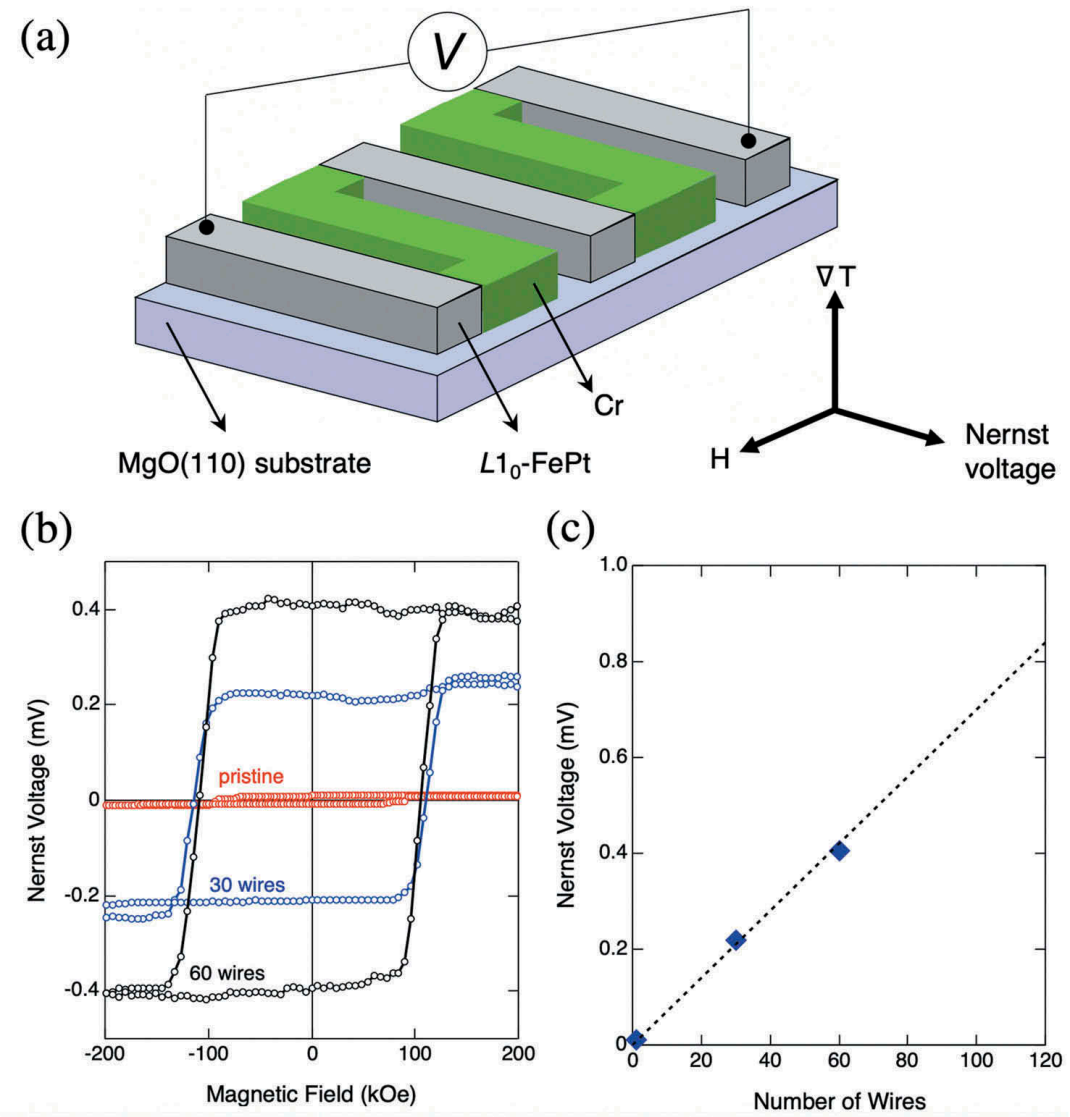
(a) The thermopile where ferromagnetic *L*1_0_-ordered FePt wires and nonmagnetic Cr wires are connected alternatively in series. Each wire has a size of 6 mm length, 5 μm width, and an interval of adjacent wires of 5 μm. An electromotive force between both edges of thermopile is measured as the Nernst voltage at room temperature with out-of-plane ∇*T* and in-plane *H*. (b) The Nernst voltage as a function of magnetic field with different numbers of FePt wires. (c) The ANE voltage as a function of the number of FePt wires [16].

An operation of the ANE-based thermopile with ∇*T* along the in-plane direction is demonstrated above. However, a device operating with ∇*T* along the out-of-plane direction is more advantageous in terms of the practical use for heat harvesting. To evidence the performance of the device with ∇*T* along the out-of-plane direction, a thermopile including FePt wires with in-plane magnetic anisotropy is fabricated and the ANE is subsequently measured. A MgO(110) substrate is employed to fabricate an FePt thin film with a thickness of 100 nm and the in-plane magnetic anisotropy of 7.0 × 10^6 ^erg/cm^3^ along FePt [001] direction. This film is microfabricated into two thermopile structures with 30 wires and 60 wires. The ANE is measured under the temperature gradient ∇*T* from surface of the film to the MgO substrate, produced by heating the bottom of the substrate. Magnetic field is applied along the easy magnetization direction in the plane, that is, FePt [001] as shown in Figure 6(a). Figure 6(b) presents the Nernst voltage measured as a function of magnetic field. Clear hysteresis loops are observed for thermopiles, and they are apparently larger than that of a pristine film, like the case for the in-plane ∇*T*. A plot between the number of the FePt wire and the Nernst voltage shows the linear relationship (see Figure 6 (c)), and the proof of the concept is made also for a perpendicular-temperature-gradient type thermoelectric device. ∇*T* and the temperature difference (Δ*T*) in the FePt layer are calculated by assuming the temperature distribution inside a MgO substrate and by employing *Q*_s_ of an FePt with the same *K*_u_. ∇*T* and Δ*T* in this thermopile are 1.0 K/mm and 1 × 10^−4^ K, respectively. These results highlight an important feature of the ANE that even extremely small temperature difference of 1 × 10^−4^ K can yield a large voltage of 0.4 mV as discussed above. The two-dimensional ANE-based thermopile structure is found to be a promising form of energy harvesting.

We have shown the possible thermopile structures. The proposed device here has an effective plane area of 6.0 × 1.2 mm^2^, and ∇*T* of 1.0 K/mm is applied normal to the plane. Thus, the generated voltage per area is estimated to be 5.6 mV/cm^2^. As shown above, the voltage increases with the number of wires, and hence we can increase the number per area by shrinking the width of each connected wire even though the resistance of the thermopile inevitably increases. Suppose that the FePt wires with a width of 100 nm and a thickness of 100 nm are connected with an interval of 100 nm. When the same temperature gradient is applied to this thermopile perpendicular to the plane, the voltage of 350 mV can be generated per square centimeter. The internal resistance of the thermopile becomes about 1 MΩ, and the output power of about 120 nW/cm^2^ can be obtained theoretically. While there may be a possibility of some unexpected heat radiation and/or electrical shortage, this output power deserves attention to an ANE thermopile for a heat-harvesting thermoelectric application.

## 5. New trends in development of prominent materials for ANE and energy-harvesting thermoelectric applications

In this section, we discuss the recent development of various materials or nanostructures revealing prominent properties regarding the ANE. In addition, an example of the realistic application of the ANE is reviewed.

**Figure 7.**
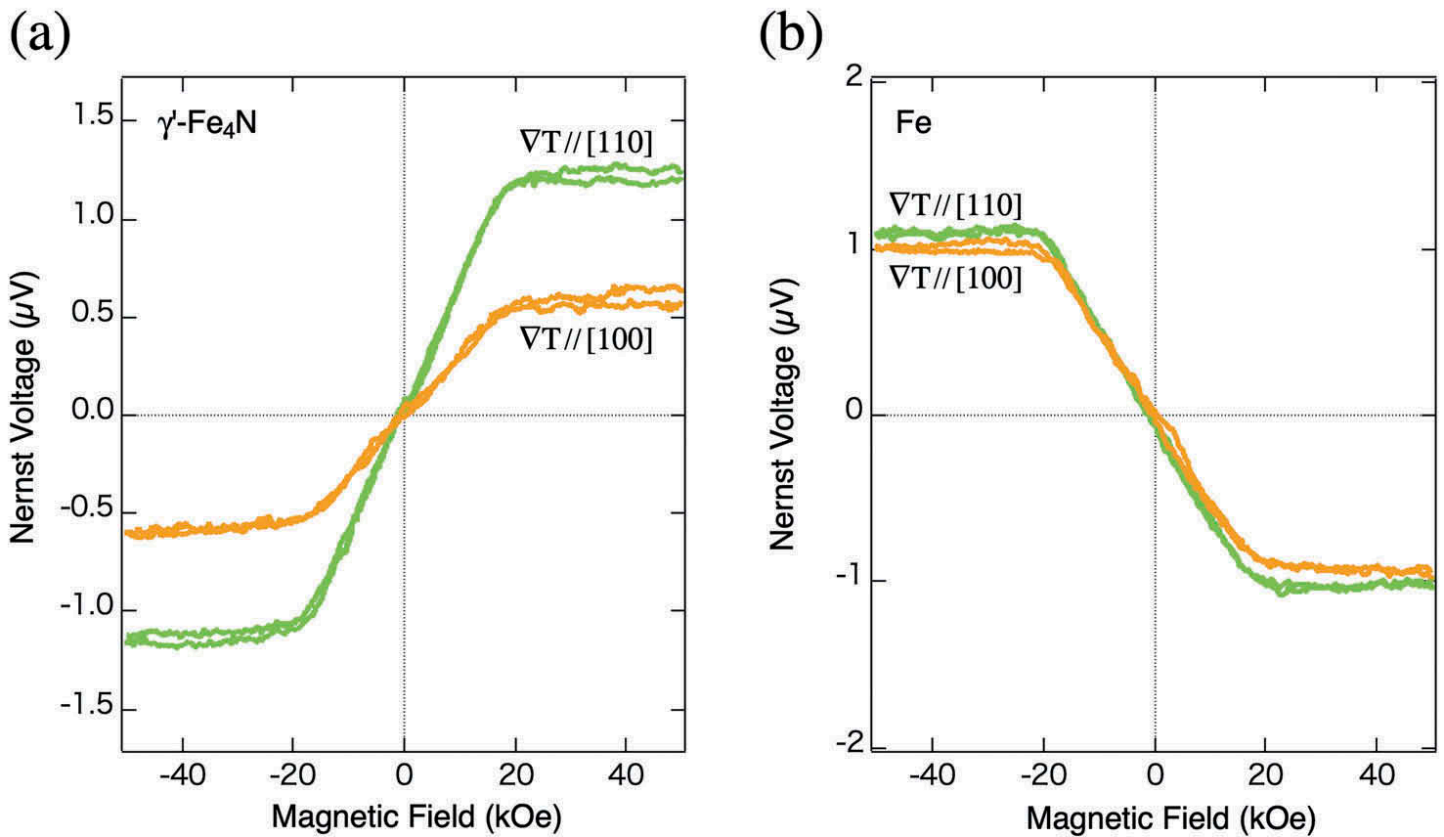
(a) The ANE voltage loops as a function of magnetic field of a *γ*’-type Fe_4_N thin film at room temperature with the two different temperature gradient directions (along Fe_4_N [110] and Fe_4_N [100] in the film plane) [18]. (b) The ANE voltage loops as a function of magnetic field of an Fe thin film at room temperature with the two different temperature gradient directions (along Fe [110] and Fe [100] in the film plane).

Some of nitride alloys are also ferromagnetic ordered alloys, and *γ*’-type Fe_4_N is one of the ferromagnetic nitride materials. This material exhibits an inverse tunneling magnetoresistance and known to have many unique characteristics [33–37]. Thus, this material is expected to have a distinctive thermomagnetic property. In this section, a highly ordered *γ*’-Fe_4_N thin film is epitaxially fabricated by a reactive magnetron nitride sputtering system, and the ANE of the film is measured [18]. Surprisingly, the anisotropy of the ANE is discovered for this material at room temperature. Figure 7(a) shows two ANE voltage loops with the two different temperature gradient directions (along Fe_4_N [110] and Fe_4_N [100] in the film plane). The Nernst voltage with ∇*T*//[110] is almost twice larger than that with ∇*T*//[100] even though the magnitude of ∇*T* (0.35 K/mm) is the same for the two configurations, and the strong anisotropy in the magnitude of the ANE is seen. Note that the Seebeck coefficients measured for the two configurations of this material are almost same. This implies that *θ*_N_ of *γ*’-Fe_4_N has strong anisotropy. As a reference, ANE voltage loops with the two different temperature gradient directions (1.47 K/mm along Fe [110] and Fe [100] in the film plane) of an epitaxial Fe thin film with a thickness of 100 nm are shown in Figure 7(b). One can observe almost no anisotropy in the Fe film as usually expected. Though the reason for a strong anisotropy in ANE of this material is not clarified yet, it is possible that the unique electronic structure of the *γ*’-Fe_4_N brings the anisotropy. Further investigation is expected for the development of nitride spintronics in the future. Anyhow, it is concluded that the effective control of the ANE can be achieved through the anisotropy.

**Figure 8.**
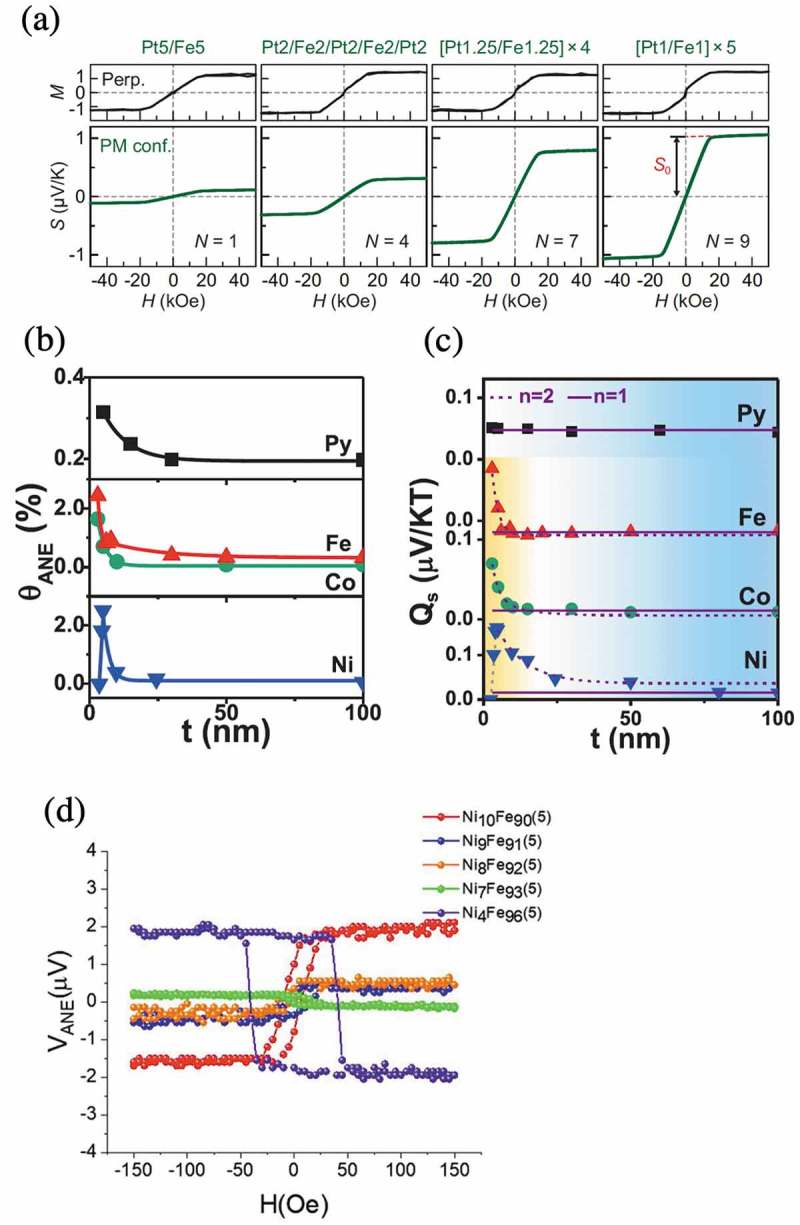
(a) The perpendicular magnetization curves and the transverse thermopower (*S*) for the Pt/Fe multilayer samples for various numbers of the interface (*N*) with in-plane ∇*T* and out-of-plane *H*. Figure adapted from Ref [19]. (b) Anomalous Nernst angle as a function of thickness of Fe, Co, Ni, and Permalloy (Py). Figure adapted from Ref [20]. (c) *Q*_s_ as a function of thickness of Fe, Co, Ni, and Permalloy (Py). Figure adapted from Ref [20]. (d) The anomalous Nernst voltage measured for Ni*_x_*Fe*_100-x_* thin films with a thickness of 5 nm. Figure adapted from Ref [21].

As discussed above, it is indispensable to control properties of the ANE such as the magnitude and sign of the ANE signal at room temperature by various means to realize ANE-based applications. A few ways of enhancing the ANE signal have been reported so far. For instance, enhancement of the ANE in metallic multilayers such as Pt/Fe, Au/Fe, and Cu/Fe are reported. The transverse thermopower (*S*) for the Pt/Fe multilayer samples increases with increasing the number of the interface even though the saturation magnetizations of the films are almost same as shown in Figure 8(a). The possibility of unconventional interface-induced thermoelectric conversion has been proposed [19]. In addition, significant enhancement of *θ*_N_ and *Q*_s_ for ferromagnetic ultrathin metal films of Fe, Co, Ni, and permalloy (Py) has been reported in a thickness-dependence study as shown in Figure 8(b,c) [20]. Thus, the establishment of methods to control the ANE is presently an essential topic. Composition dependence of ANE is also studied for NiFe thin films [21]. Figure 8(d) shows the anomalous Nernst voltage measured for Ni*_x_*Fe*_100-x_* thin films with a thickness of 5 nm. They claim that the competition between opposite anomalous Nernst signals of constituent elements are invoked by changing the composition, and the Nernst voltage decreases to almost zero in Ni_7_Fe_93_.

**Figure 9.**
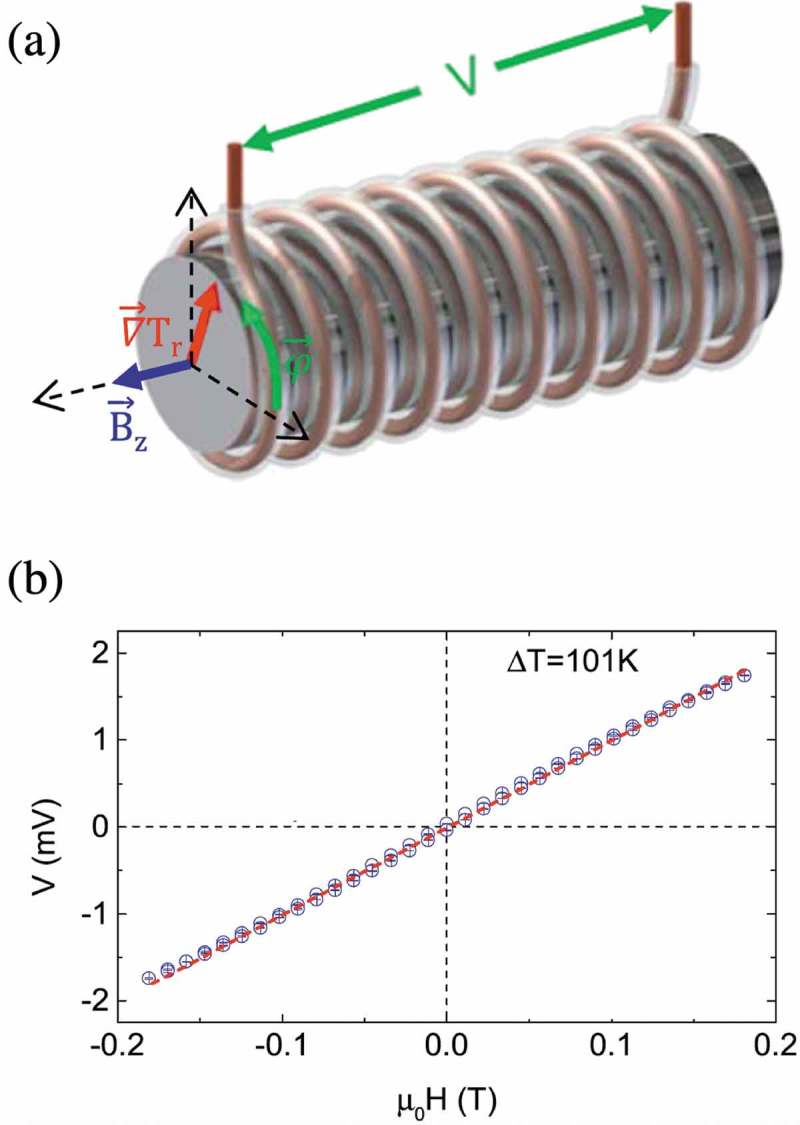
(a) A schematic image of scalable generation of a Nernst voltage in an air-cooled metal wire coiled around a hot cylinder. A radial temperature gradient generates an azimuthal Nernst electric field in the coil. A Galfenol wire is wrapped around a cartridge heater and a temperature difference of 101 K is applied. Figure adapted from Ref [22]. (b) The Nernst voltage measured for an air-cooled metal wire coiled around a hot cylinder as a function of magnetic field. Figure adapted from Ref [22].

Moreover, scalable generation of a Nernst voltage in an air-cooled metal wire coiled around a hot cylinder has been recently reported as shown in Figure 9(a) [22]. A radial temperature gradient generates an azimuthal Nernst electric field in the coil. A Galfenol wire is wrapped around a cartridge heater and a temperature difference of 101 K is applied. The generated Nernst voltage is measured as a function of axial magnetic field. As expected, the Nernst voltage increases linearly with the magnetic field as shown in Figure 9(b). These recent developments suggest that a thermoelectric device based on the ANE that can operate at room temperature will consequently be realized in the near future.

## 6. Large ANE observed in the Weyl antiferromagnet: Mn_3_Sn

The recent Berry phase formulation of the transport properties has led to the discovery that a large anomalous Hall effect (AHE) may arise not only in ferromagnets but also in antiferromagnets and spin liquids, in which the magnetization is vanishingly small [38–44]. As the first example in antiferromagnets, one of the authors has discovered that Mn_3_Sn exhibits a large AHE [42]. Theoretically, the AHE is obtained by an integration of the Berry curvature for all the occupied bands, and the ANE is determined by the Berry curvature at *E*_F_ [45,46]. Thus, the observation of a large AHE does not guarantee the observation of a large ANE. At the same time, the ANE measurement should be highly useful to clarify the Berry curvature spectra near *E*_F_ and to characterize the Weyl metal recently found for Mn_3_Sn [47,48].

**Figure 10.**
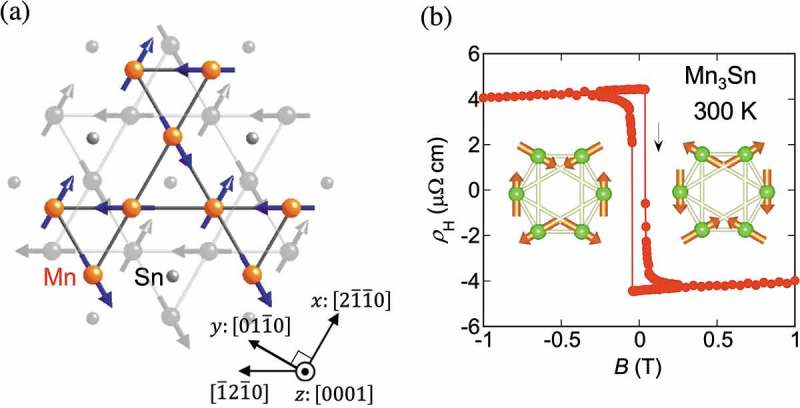
(a) Unit cell crystal structure of Mn_3_Sn. To distinguish Mn and Sn on different *xy*-planes with *z* = 0, 1/2, those atoms in different planes are shown by different colors. Mn atoms form a breathing type Kagome lattice, and their spins have a 120-degree magnetic structure as shown by blue arrows. Here, we define [2-1-10], [01-10], and [0001] as *x, y*, and *z* axes, respectively. (b) Field dependence of the Hall resistivity *ρ*_H_ obtained at 300 K in the magnetic field *B* [2-1-10] and [01-10] with the electric current *I*//[0001]. Inset: the schematic view of the cluster multipole. The direction of all the Mn moments, and thus the octupolarization, changes upon the magnetization reversal.

Mn_3_Sn has a hexagonal crystal structure with space group of *P*6_3_ */mmc* [49] (Figure 10(a)). Mn atoms form a kagome lattice in the *ab*-plane, and each Mn triangle on the kagome lattice is stacked on top along the *c*-axis. On cooling below the Néel temperature of 430 K, Mn magnetic moments of ~ 3*µ*_B_ lying in the *ab*-plane form a coplanar, 120-degree spin structure characterized by *Q *= 0 wave vector [50,51]. This structure is characterized by the negative sign of the vector chirality and called the inverse triangular spin structure, and stabilized by the combination of the geometrical frustration and Dzyaloshinskii–Moriya interactions (Figure 10(a)) [50–52]. Interestingly, this magnetic structure can be viewed as a ferroic (*Q *= 0) order of a cluster magnetic octupole shown in the inset of Figure 10(b), and thus breaks the time-reversal symmetry [53]. This symmetry breaking enables the observation of the Kerr effect in the antiferromagnetic metal [23]. In addition, it further induces a very tiny magnetization ~ 2 m *µ*_B_/Mn, allowing us to switch the non-collinear antiferromagnetic structure by using magnetic field.

Figure 10(b) shows the field dependence of the Hall resistivity *ρ*_H_(*B*) at 300 K. A sharp jump is seen in *ρ*_H_(*B*) with a small coercivity of ~ 0.01 T. The size of the jump ∆*ρ*_H_ ~ 9 µΩcm is large and is equivalent to an ordinary Hall effect under ~ a few 100 T for free conduction electrons with density of order one electron per Mn atom. The sign change in the Hall effect as a function of field indicates the rotation of the sublattice moments accompanied by the flipping of the tiny uncompensated moment [42,50,52].

**Figure 11.**
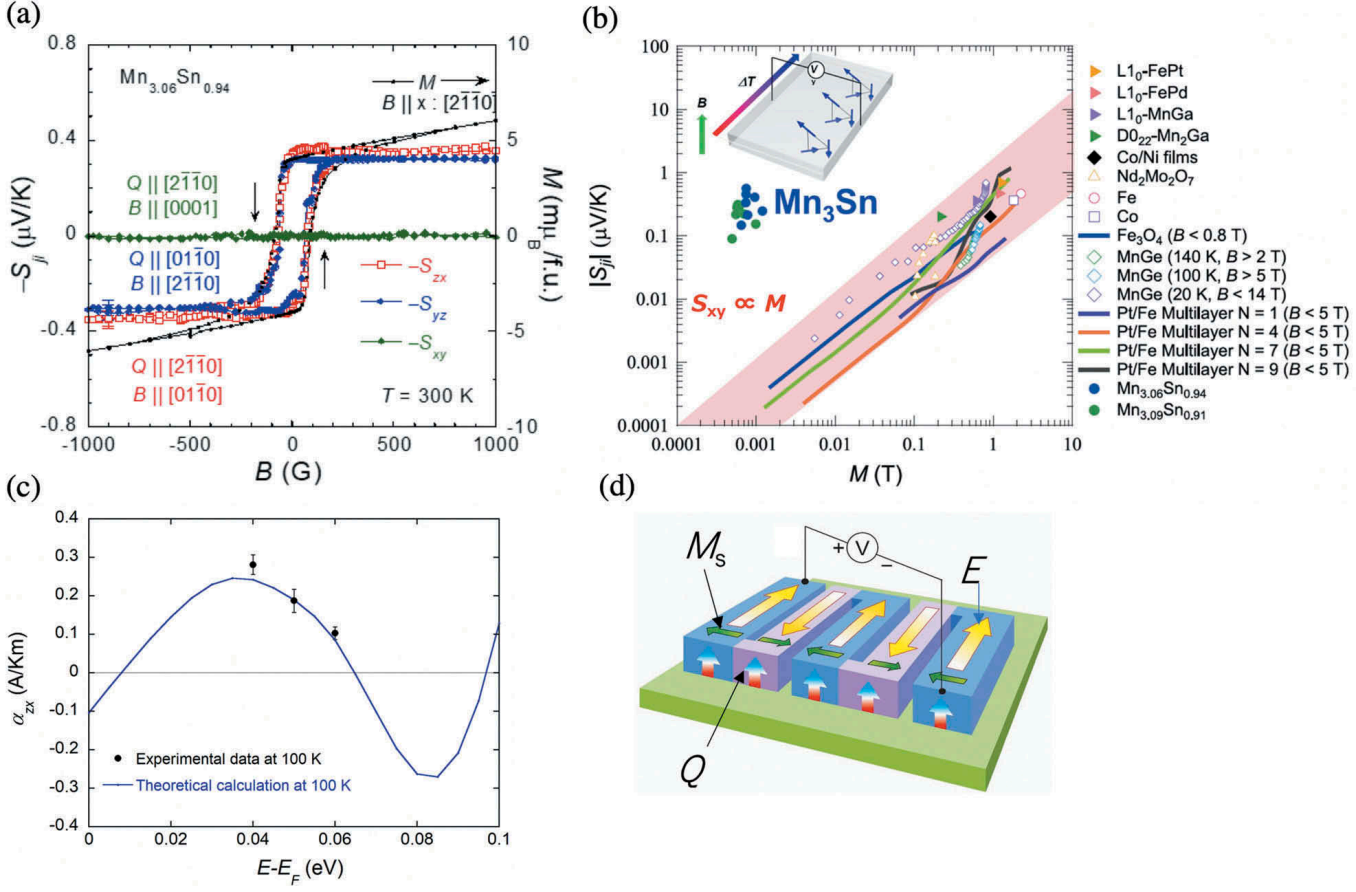
(a) Anisotropic field dependence of the Nernst signal *S*_ji_ (left axis) obtained at 300 K in the magnetic field B [2-1-10] (square), [01-10] (circle) and [0001] (diamond). For comparison, the field dependence of the magnetization *M* (right axis) for *B*//[2-1-10] is shown [7]. (b) Double-logarithmic plot of the anomalous Nernst signal |*S*_ji_| vs. the magnetization *M* for a variety of ferromagnetic metals and Mn_3_Sn measured at various temperatures and fields. It shows the general trend for ferromagnets that |*S*_ji_| increases with *M*. The shaded region indicates the linear relation |*S*_ji_| = |*Q*_s_|*µ*_0_ *M*, with |*Q*_s_| ranging from 0.05 µV/KT to 1 µV/KT. The Nernst signal data points for Mn_3_Sn obtained at various temperatures for *B*//[2-1-1 0] (red square) and [01-10] (blue circle) do not follow the relation, and reach almost the same value as the largest among ferromagnetic metals with three orders of magnitude smaller *M* [7]. Inset: The anomalous Nernst electric field *E* appears in the direction of the outer product of the magnetization *M* and heat current *Q* ~ -∇*T*. (c) Relation between *α*_xy_ and *E-E*_F_. (d) Schematic figure of a thermopile. The in-plane magnetization directions of neighboring thermoelectric modules are flipped so that the Nernst signal with the same sign can be added up in series. The heat flows along the direction perpendicular to the basal plane of the heat source [7].

Strikingly, we find that Mn_3_Sn exhibits a large ANE at room temperature as shown in Figure 11(a) [7]. The Nernst signal (transverse thermopower) *S*_ji_ exhibits a clear and sizable hysteresis. The change of ∆*S*_ji_ ~ 0.7 µV/K as a function of the in-plane field is significantly large and comparable to the values reported for ferromagnets. As shown in Figure 11(a), we find that the in-plane Nernst signal exhibits hysteresis with a small anisotropy, while the out-of-plane *c*-axis component is zero within experimental accuracy, indicating no spontaneous effect in this direction.

Conventionally, the ANE is known to be proportional to the magnetization *M*. Thus, here we compare the ANE with the magnetization *M* and plot in Figure 11(a) both *S*_ji_ and *M* as a function of the in-plane field [7]. In low fields, both data overlap on top of each other. In the higher field region than the coercivity of 100 ~ Oe, however, the ANE remains constant, unlike *M* linearly increasing with field. This field independence of the ANE indicates that the single-domain antiferromagnetic state has a large spontaneous Nernst signal that does not follow the conventional mechanism for ferromagnets where the ANE scales with *M*.

The qualitative difference between the ANE observed in Mn_3_Sn and in ferromagnets can be found clearly in a double-logarithmic plot of the anomalous Nernst signal vs. the magnetization for various ferromagnetic metals and Mn_3_Sn (Figure 11(b)) [7]. Similarly to AHE [38], the ANE for ferromagnets is known to be proportional to magnetization. Indeed, Figure 11(b) roughly confirms such an overall trend for a broad range of ferromagnetic metals. The shaded region which covers all the data indicates that the anomalous Nernst signal is indeed proportional to the magnetization *M*, with the *Q*_s_ ranging between 0.05 and 1 µV/KT. Based on this relation, Mn_3_Sn would have produced the Nernst signal of the order of 0.01 ~ 2 nV/K with the observed magnetization. Strikingly, however, *S*_xy_ ~ 0.35 µV/K found at room temperature is more than 100 times larger than what would be expected based on the above scaling relation for ferromagnets.

The large anomalous Nernst and Hall effects in Mn_3_Sn are unexpected according to their conventional scaling law with *M*, and thus should arise from the mechanism distinct from the conventional one for ferromagnets [7]. The anomalous Hall conductivity is the measure of the sum of the Berry curvature for all the occupied bands. On the other hand, ANE, or more precisely, the transverse thermoelectric conductivity *α*_xy_ is determined by the Berry curvature around the Fermi level [45,46]. Therefore, the large *α*_xy_ means that the Berry curvature is significantly enhanced at *E*_F_. In fact, a recent first-principles calculation has confirmed the Weyl points nearby *E*_F_ [47]. While it is theoretically expected that when the Weyl points locate exactly at *E*_F_, the ANE is small as the Hall effect becomes maximized with (∂*σ*_H_/∂*E*)*_E_*_F_ = 0, a slight tuning of the Fermi energy away from the Weyl point may enhance the ANE significantly (Figure 11(c)). The calculated anomalous transverse thermoelectric conductivity is found as large as seen in experiment, and has a peak with different signs around the Weyl point at *E* = 60 meV away from *E*_F_ (Figure 11(c)). Our observation of the dramatic change in the large anomalous Nernst as a function of the Fermi energy, which is fully consistent with theory, supports the idea that the Weyl points play a major role in their mechanism. Our results thus indicate that the ANE in Mn_3_Sn is particularly enhanced because of the characteristic structure of the Berry curvature with several Weyl points nearby the Fermi level [47,54]. Further developing the concept of application of Weyl magnet for enhancing ANE, we have recently found that a magnetic Weyl semimetal in the vicinity of the Lifshitz transition between Type-I and Type-II Weyl fermion states would lead to a giant ANE. In fact, the Weyl ferromagnet Co_2_MnGa is found to exhibit a record high ANE of 6 μV/K at room temperature, one order magnitude larger than the ordinary ferromagnet [26].

Finally, for thermoelectric power generation, ANE should be useful as it enables the fabrication of a thermopile module structurally much simpler than the conventional Seebeck device as discussed above. As the voltage output appears in the perpendicular orientation to the thermal heat flow (Figure 11(b) inset), we may cover the heat source with a lateral series connection of a single kind of ferromagnet with alternating magnetization direction (Figure 11(d)). This configuration enables a thermopile structure to efficiently cover the surface of a heat source. The antiferromagnetic thermoelectric material would be particularly useful in comparison with a ferromagnetic counterpart, as it has no inherent stray fields that may perturb magnetization direction of neighboring modules, and enhances the integration density which leads to the advanced energy-harvesting system.

## 7. Summary

Various topics related to the basic understanding and modulation of the ANE and new materials generating a large ANE are discussed including the recently discovered Weyl magnets. The ANE is essential for investigating the interplay among heat, spin, and charge in magnets. In addition, compared to the Seebeck effect, it has various benefits for application to high-efficiency energy-harvesting devices as it may provide much more simple lateral structure, higher flexibility, and much lower production cost. The possible methods for thermoelectric application of the ANE are reviewed, providing basic strategies for the modulation of the ANE and for effectively utilizing the ANE in the energy-harvesting technology.
